# Revascularization of an Immature Permanent Central Incisor with Complicated Crown Root Fracture: A Case Report

**DOI:** 10.5005/jp-journals-10005-1574

**Published:** 2019

**Authors:** Anu John, Amitha M Hegde, Preeth Shetty, Shreema Shetty

**Affiliations:** 1–4 Department of Pedodontics and Preventive Dentistry, AB Shetty Memorial Institute of Dental Sciences, Mangaluru, Karnataka, India

**Keywords:** Crown root fracture, Immature teeth, Revascularization

## Abstract

The treatment of complicated crown root fracture in children possesses a great challenge to pedodontists. A 10-year-old boy presented with trauma to the permanent maxillary left central incisor. Upon clinical and radiographic examination, revealed mobility of coronal fragment, open apex, and vertical fracture involving enamel dentin and pulp. Instead of the standard root canal treatment protocol, revascularization was performed after stabilizing the coronal fragment. The detachment of the coronal fragment occurred following a fall for the second time after 1 month of placement of mineral trioxide aggregate (MTA) over the blood clot. The coronal seal was intact; hence, the coronal fragment was reattached and the crown was placed. At 12-month follow-up, clinical and radiographic evaluation showed that the tooth was asymptomatic and functional. With increasing scope of clinical facts, patient, and practitioner favorable reception, regenerative techniques may be used as a standard technique in treating complicated crown root fracture in young permanent teeth.

**How to cite this article:**

John A, Hegde AM, *et al.* Revascularization of an Immature Permanent Central Incisor with Complicated Crown Root Fracture: A Case Report. Int J Clin Pediatr Dent 2019;12(1):59–63.

## INTRODUCTION

The dental treatment for avulsed or traumatized teeth with an exposed pulp can vary considerably. Some dentists debride the pulp tissues and obturate the root canal with gutta-percha,^1–4^ composite resin, calcium hydroxide,^5^ or MTA.^6^ Meanwhile, other dentists will attempt regenerative endodontic procedures to restore the vitality of a tooth. Regenerative endodontic procedures involving pulp capping, partial pulpotomy, and root canal revascularization procedures have also been used since the 1970s.^7,8^

About 96% of endodontists are willing to include regenerative therapies into treatments and the practitioner interest to carry out regenerative endodontic procedures has been growing.^9^

Regenerative endodontic procedures can be defined as biologically based procedures that predictably replace damaged, diseased, or missing structures including dentin and root structures and cells of the pulp–dentin complex with viable tissues of the same origin that restores the normal physiologic functions of the pulp–dentin complex.^10^

Traumatized immature teeth could benefit from regenerative endodontic procedures such as revascularization, apexogenesis, and partial pulpotomy.

The purpose of this case report is to present a case of pulp revascularization in an immature permanent tooth with complicated crown root fracture.

## CASE DESCRIPTION

A 10-year-old patient presented to the Department of Pedodontics and Preventive Dentistry for evaluation and treatment of maxillary left central incisor (tooth 21). The patient was accompanied by his mother and reported that her son had suffered dental trauma due to fall from the bicycle 1 week back. No fracture of the tooth was noted by the parents after the fall. Parents reported that their son developed pain 2 hours after the accident in the upper front tooth region with difficulty in biting.

On the same day, the patient visited the general dentist with the same complaint and was prescribed antibiotics and analgesics. The patient was asked to report after 1 week for evaluation. The patient reported to our department for further treatment.

The medical history of the patient was noncontributory and the patient had no prior dental visit to the dental school.

**Fig. 1:**
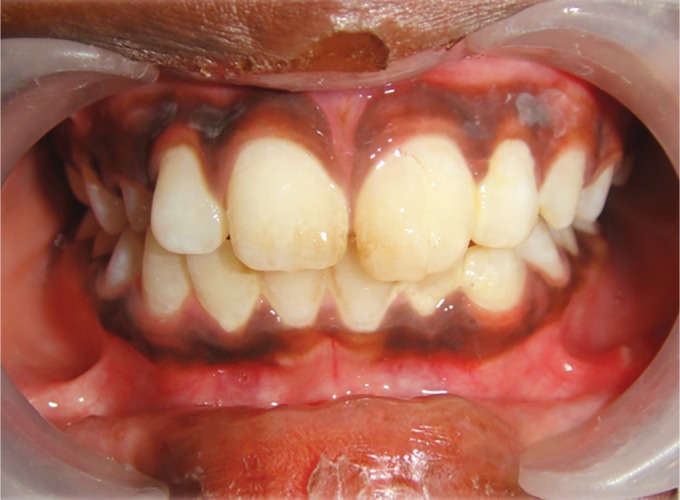
Patient's anterior tooth no. 21 showing vertical fracture and step deformity of the coronal fragment

Clinical examination revealed (1 week after trauma) a vertical fracture line extending from the incisal third to the cervical of the crown (tooth no. 21) and the fractured segment appeared to be mobile on palpation and periodontal probing showed 3 mm in depth (Fig. 1).

The diagnostic test was inconclusive on cold and electric pulp testing, but the patient reported sensitivity to percussion and palpation.

**Fig. 2:**
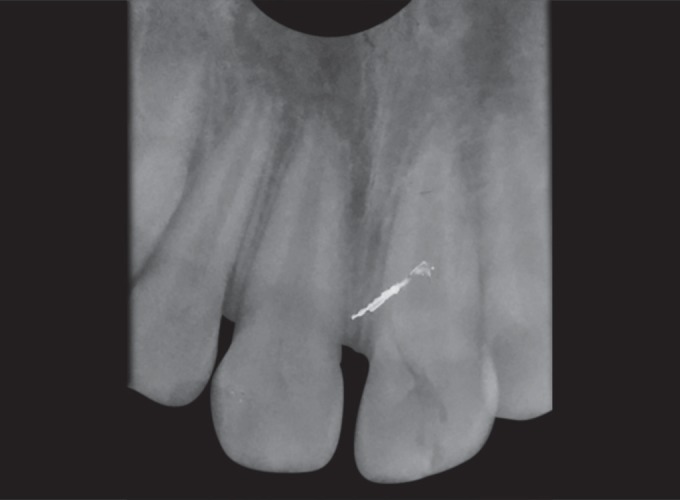
Periapical radiograph of the permanent left central incisor showing open apex, vertical fracture involving enamel, dentin, and pulp and extending up to 3 mm above the cervical line

Radiographic examination revealed open apex and there were no signs of periapical pathology and it was noted that the vertical fracture extended from the mesial thirds of the crown involving enamel, dentin, pulp, and extending up to 3 mm above the cervical line (Fig. 2).

A diagnosis of a complicated crown root fracture was made for tooth no. 21.

The mother was informed about the limitations and advantages of revascularization as a treatment modality, and informed consent was obtained.

**Fig. 3:**
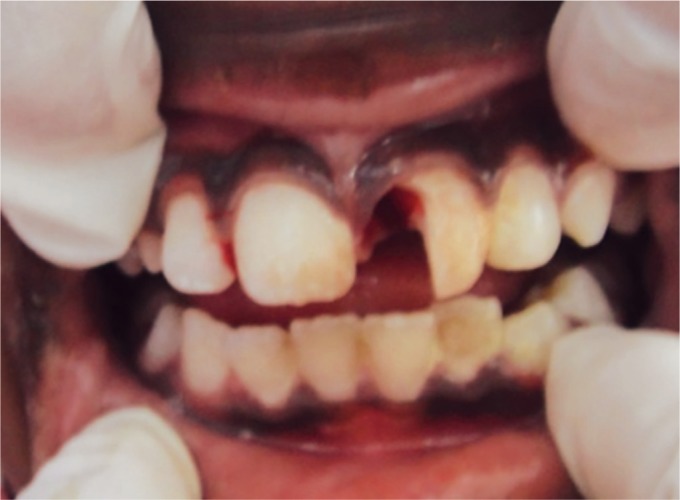
Patient's anterior tooth on the removal of the coronal fragment

**Fig. 4:**
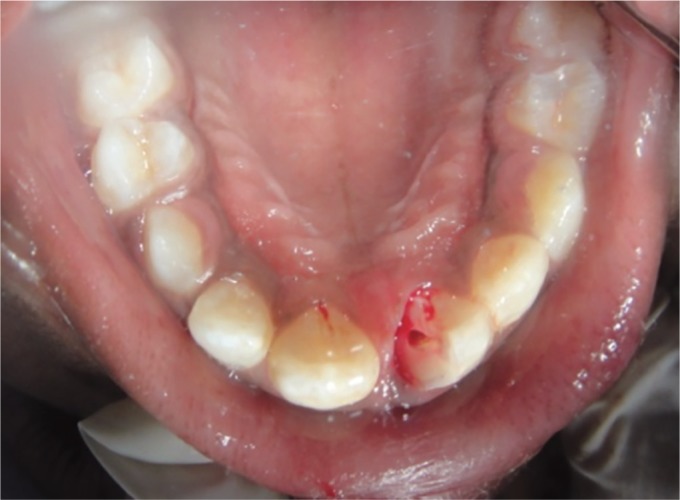
Patient's anterior tooth on the removal of the coronal fragment (posterior view)

After administration of local anesthesia with 1.8 mL of 2% lidocaine with 1:100,000 epinephrine, the fractured segment was carefully removed using forceps and was kept under saline solution. On removal, there was a direct access to the pulp chamber without advocating an access cavity preparation and bleeding from the canal was seen (Figs 3 and 4).

Complete extirpation of the radicular pulp was advocated and the root length was estimated radiographically using a size 30-K file.

To facilitate revascularization procedure, the coronal third of the root canal was widened passively with Gates Glidden drill size ≠ 4 (Dentsply Maillefer, Tulsa, OK, USA). The canal was passively irrigated with 10 mL of 5.25% sodium hypochlorite solution (NaOCl) for 15 minutes and followed by 20 mL saline then dried with absorbent points (Dentsply Maillefer).

**Fig. 5:**
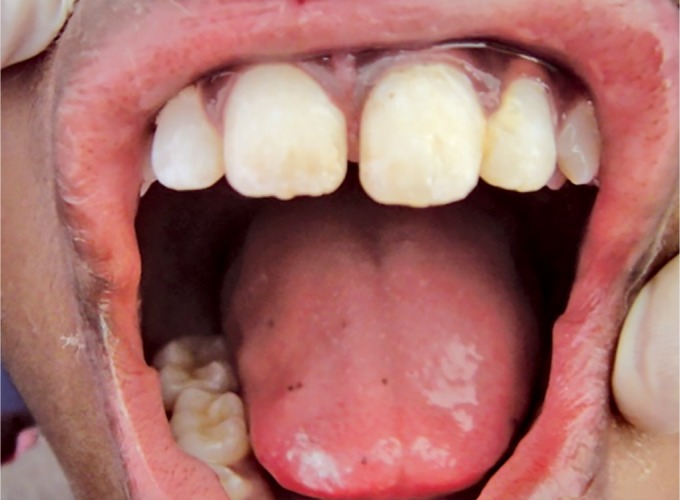
Patient's anterior tooth (no. 21) after stabilizing the coronal fragment

**Fig. 6:**
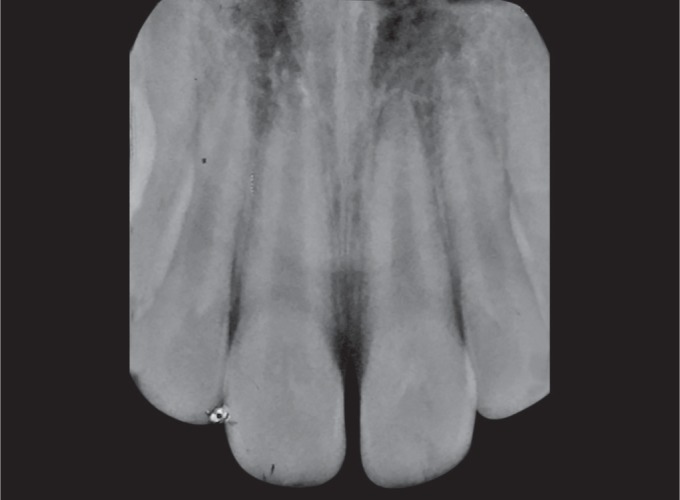
Periapical radiograph taken after stabilizing the coronal fragment

Ca(OH)_2_ paste (powder mixed with saline, Merck, Frankfurt, Germany) was placed into the canal, covered with a sterile cotton pellet, and temporized using an intermediate restorative material (Caulk Dentsply, Milford, DE). The fractured segment was carefully approximated with the help of fingers and repositioned and stabilized (Fig. 5) using the light cure glass ionomer cement (Fuji II Light cure Glass ionomer cement, GC Corporation, Tokyo, Japan). A radiograph was taken immediately and a close approximation of the fractured segment was observed (Fig. 6).

The patient returned after 1 week and was asymptomatic, under local anesthesia and the rubber dam isolation pulp chamber was accessed from the palatal surface. The temporary restoration was removed. The root canal was slowly flushed with 15 mL of 5.25% NaOCl for 15 minutes and followed by 20 mL of saline solution. The canals were dried with absorbent points.

**Fig. 7:**
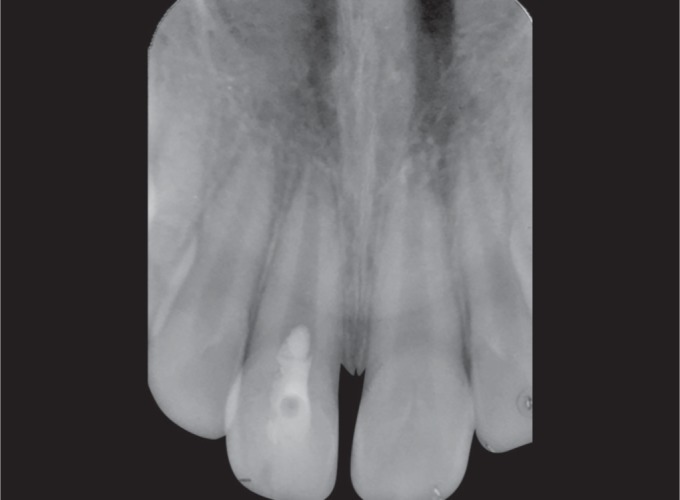
Mineral trioxide aggregate placement at the cervical level after the blood clot was formed

**Fig. 8:**
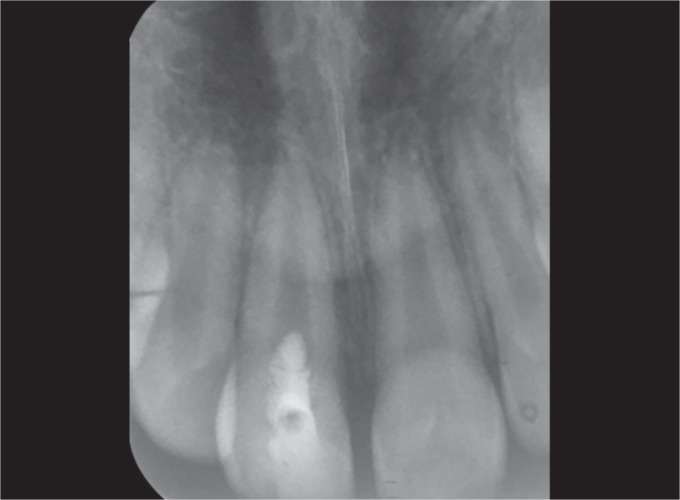
3-month follow-up showing early stages of apexogenesis

**Fig. 9:**
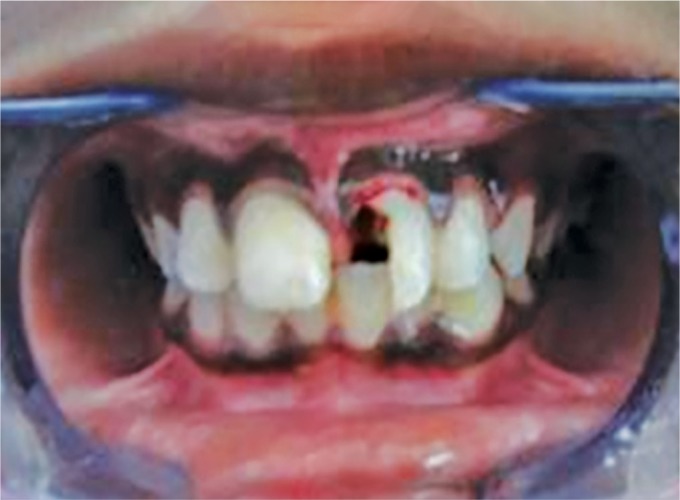
Detachment of coronal fragment after the second fall (anterior view)

A freshly prepared antibiotic paste consisting of ciprofloxacin, metronidazole, and minocycline (100 mg of each drug in 0.5 mL total volume) was placed into the canal using lentulo spiral (Dentsply Maillefer), by keeping 2 mm short of the working length and was covered with a sterile cotton pellet followed by the placement of intermediate restorative material and the cavity was sealed with the glass ionomer cement (Fuji II, GC Corporation, Tokyo, Japan). The patient was asked to report after 3 weeks.

At 3 weeks, the patient was asymptomatic, and the tooth showed no tenderness to percussion and palpation. Under local anesthesia and rubber dam isolation, the triple antibiotic paste was removed using 15 mL of 5.25% NaOCl, for 15 minutes and followed by 20 mL of saline solution. The canals were dried with absorbent points. A size #30 K-file was used and introduced into the apical area to initiate bleeding into the canal space. The bleeding was left for 15 minutes, so that blood would clot. After 15 minutes, MTA (Dentsply) was carefully placed over the blood clot followed by a wet cotton pellet and an intermediate restorative material was placed and sealed with the glass ionomer cement (Fig. 7).

At the third month recall, the patient was asymptomatic, and the radiograph showed normal periodontal findings (Fig. 8). Glass ionomer cement, intermediate restorative material, and cotton pellet were removed and replaced with the bonded resin restoration (Filtek A110; 3M Dental Products, USA).

**Fig. 10:**
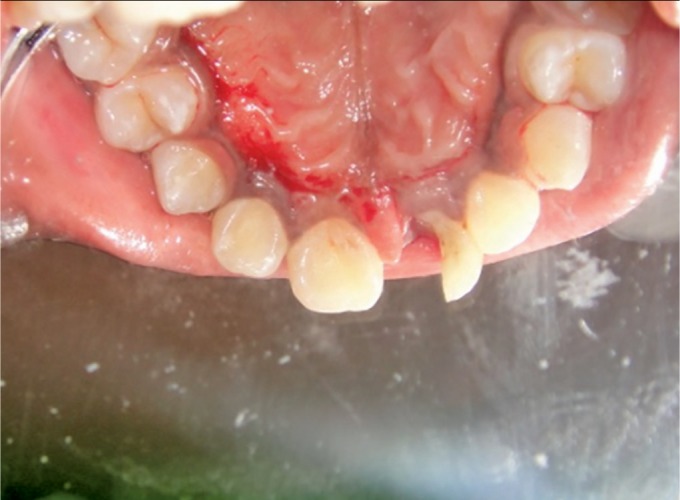
Detachment of coronal fragment after the second fall (posterior view)

Ten days later, the patient had a fall again and reported to the department after 3 hours following trauma with the detachment of the coronal fragment which was stabilized. Clinically, the access cavity was seen intact and covered with resin restoration (Figs 9 and 10).

The patient was asymptomatic, and the tooth showed no tenderness to percussion and palpation. The detached crown fragment was repositioned and stabilized using the light cure glass ionomer cement.

**Fig. 11:**
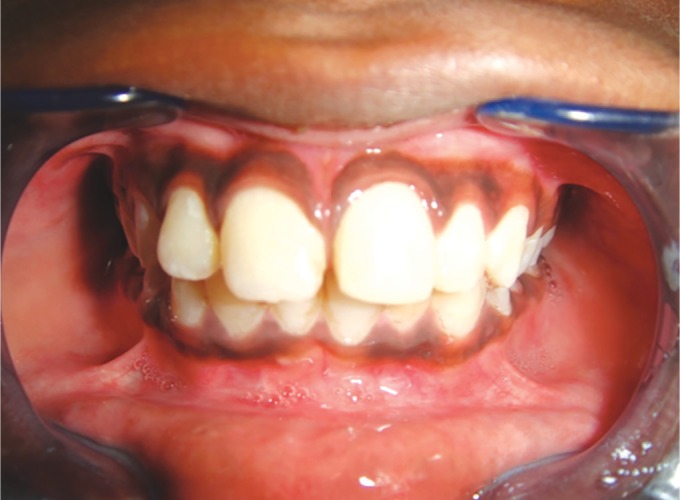
Acrylic crown cemented on tooth no. 21

On the follow-up appointment, tooth preparation was done and the temporary crown was cemented. The acrylic crown was delivered after 1 week (Fig. 11) and the patient was informed that acrylic crown would be replaced with the permanent crown if the tooth became symptomatic at a later stage, then would require endodontic intervention.

**Fig. 12:**
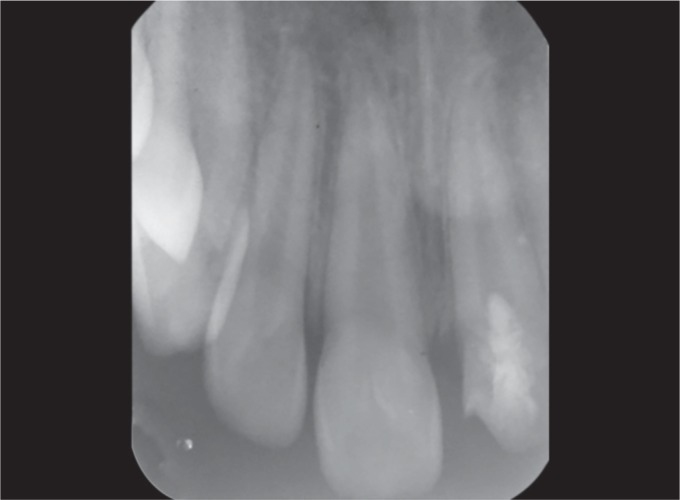
6-month follow-up showing continued apexogenesis

**Fig. 13:**
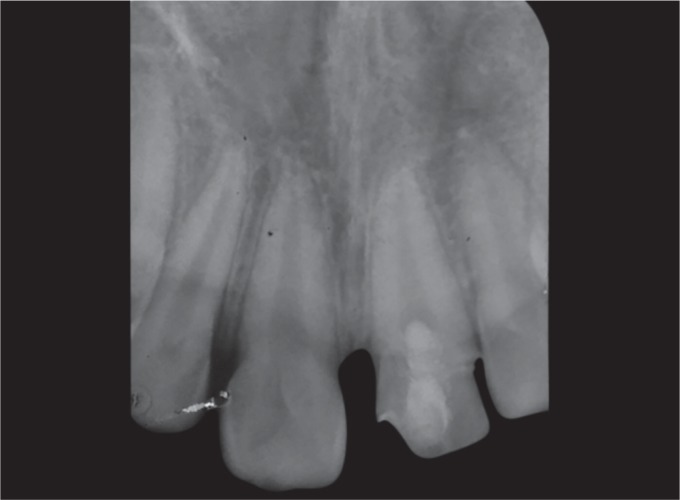
1-year follow-up showing continued apexogenesis and dentinal wall thickening

At 6 months and at 1 year of follow-up, the tooth continued to be asymptomatic and functional. Apical closure and dentinal wall thickening were evident along with the attainment of root length (Figs 12 and 13).

## DISCUSSION

This case report describes a revascularization procedure in a complicated crown root fracture, demonstrating the successful outcome of the regenerative procedure.

The treatment of young permanent tooth with an open apex often presents a unique challenge to the dentist. In this case, the patient presented with complicated crown root fracture with mobility of the coronal fragment. According to the International Association of Dental Traumatology (IADT), the guideline for the management of complicated crown root fracture describes the stabilization of the coronal fragment, endodontic procedure, and post-retained crown.^11^

However, in this case, the coronal fragment was stabilized and revascularization procedure was done.

A detachment of the coronal fragment following the second fall after placement of MTA over the blood clot would have compromised the outcome of the treatment. The importance of bacterial tight coronal seal is well documented for the success of the regenerative procedure.^12^ The patient reported with an intact coronal seal and, hence, the detached fragment was repositioned and stabilized using the light cure glass ionomer cement.

After 5 days, crown preparation was done and the acrylic crown was placed. At the 6-month follow-up and after 1 year of reinitiating, the patient was asymptomatic and the tooth was functional and showed no tenderness to percussion and palpation. Periodontal probing was within physiologic limits. Thickening of the root walls and formation of the apical barrier was seen radiographically.

This treatment approach was seen to be beneficial to the patient in maintaining the functionality and vitality of the traumatized tooth over the conventional endodontic procedure.

## CONCLUSION

Revascularization technique is a less invasive procedure for children presenting with complicated crown root fracture in the young permanent tooth with an open apex. This treatment is technically sensitive and challenging; hence, regular follow-up for clinical and radiographic evaluation is highly recommended for the success of the revascularization procedure.
